# When investigating depression and anxiety in undergraduate medical students timing of assessment is an important factor - a multicentre cross-sectional study

**DOI:** 10.1186/s12909-020-02029-0

**Published:** 2020-04-23

**Authors:** Pia Thiemann, James Brimicombe, John Benson, Thelma Quince

**Affiliations:** The Primary Care Unit, Department of Public Health and Primary Care, University of Cambridge, Cambridge, UK

**Keywords:** Depression, Anxiety, Undergraduate medical students, Timing of assessment, Final exams

## Abstract

**Background:**

Symptoms of depression and anxiety experienced by undergraduate medical students have become a prominent concern. Evidence about students’ depression and anxiety including prevalence, trajectory during medical education, gender differences and comparisons with age-matched peers is conflicting. However few studies of medical students’ mental health specify the precise time of assessment. Proximity to examinations may be relevant. Precise identification of the time of data collection might help explain contradictory findings and facilitate provision of more timely support.

**Methods:**

This study addressed whether:

1. Proximity of final examinations affected students’ depression and anxiety symptoms
2. Males and females differed in this respect.

We analysed data provided by 446 final year students from 6 UK medical schools. These students were a subset of data provided by 14 UK medical schools which participated in an online survey comparing first and final year students and in which final year response rates exceeded 30%. We used the Hospital Anxiety and Depression Scale to assess symptoms of depression and anxiety and the norms to indicate potentially clinically relevant cases. We grouped students into those for whom final exams were imminent i.e. within 2 months of completing the survey (*n* = 164) and those for whom exams were more distant or had been taken (*n* = 282). We used parametric and non-parametric tests to compare both groups and gender differences in respect of depression and anxiety sum scores and cases rates.

**Results:**

For both depression and anxiety male and female students facing imminent final exams recorded greater prevalence and significantly higher mean scores. The effect size of differences for anxiety were large. No substantial gender differences were found for depression. Regardless of the timing of final exams female students recorded both significantly higher mean scores and clinically relevant rates for anxiety.

**Conclusions:**

Proximity to final exams negatively affected the mental health of both male and female final year students. The study suggests that there may be times in the undergraduate medical curriculum when additional or targeted support is needed. It also highlights the need for research to provide a greater specificity of context when investigating medical students’ mental health.

## Background

Although an issue of concern for many years, symptoms of depression and anxiety experienced by undergraduates, and in particular medical students, have become the focus of recent research [1, 2]. Concern is not just for the possible impact on achievement during medical school but also, potentially, on professional commitment and future patient care [3–6]. A systematic review encompassing studies of medical students in Europe and the English-speakingworld outside North America reported prevalence ranging from 6.0 to 66.5% for depression and from 7.7 to 65.5% for anxiety [7]. The authors also noted the relatively poor quality of studies, the use of different validated instruments which may not be entirely comparable, failure to report the cut-offscores used to indicate severity or otherwise of depression or anxiety and the use of varying cut-offscores for validated instruments. They indicated that better quality studies tended to report lower prevalence [7].

Other mental health issues have been found to affect medical students. Studies in several countries have found relatively high levels of substance abuse (both alcohol and drugs) and both eating and sleep disorders [8–10]. The focus of this paper is on the symptoms of depression and anxiety experienced by medical students. Studies of undergraduate medical students, undertaken in different countries, suggest that students experience increasing mental health issues including symptoms of depression and anxiety as their course progresses [11, 12]. However other studies have found higher levels of depression and anxiety among medical students at the outset of their course [13–15]. Unfortunately most studies have been cross-sectionaland where longitudinal studies have been undertaken, they are generally based in a single institution making generalisation difficult [16, 17].

Evidence that female medical students display higher levels of depression and anxiety than their male counterparts is similarly mixed. Studies in many countries have reported higher levels of symptoms among female medical students compared to males [13, 18]. For example female students in Serbia and Pakistan were found to record significantly higher levels of both depression and anxiety but gender differences for anxiety were more pronounced than those for depression [18, 19]. By contrast, a study of Chinese medical students found a significantly higher proportion of male students had symptoms of depression compared to female students [12].

Research has also suggested that the demands of medical education mean that medical students are more susceptible to depression and anxiety compared to non-medicalstudents and non-studentage matched peers [20]. However a review comparing the prevalence of depressive symptoms among medical and non-medicalstudents concluded that medical students had similar or lower rates of depression compared to certain groups of non-medicalstudents and there are few studies which have compared undergraduate medical students with age matched peers [21].

It is notable that studies examining the trajectory of symptoms of depression and anxiety amongst medical students generally omit to describe the precise study points being compared. There is also evidence that students are more likely to experience symptoms of depression and anxiety at stressful times such as close to examinations [22–24]. Brand and Schoonheim-Klein(2009) examined the association between state anxiety displayed by dental students and different assessment methods, they found all types of assessment raised state anxiety but OSCEs were found to be particularly stressful [25].

Evidence suggests that medical students are particularly reluctant to seek help for mental health issues [26–29]. Better identification of the precise times at which medical students are more likely to be vulnerable can help to facilitate better support. Better identification of precise times may also facilitate clarification of conflicting research findings.

In a previous longitudinal study in one UK medical school, we followed the same undergraduate medical students through their course, examining the prevalence and trajectory of depression of four cohorts at 3 time points [17]. We used the Hospital Anxiety and Depression Scale (HADS-D) [30]. Prevalence rates of raised levels of depression (HADS-D scores of ≥8) ranged from 2.7–10.6% and did not increase over time. We found a statistically significant increase overtime in mean scores for depression among male students only but this difference was small in terms of effect size as measured by Cohen’s D [17, 31]. However very few students repeatedly recorded scores indicating raised symptoms of depression. We concluded that for many students, depression may be transitory, and for many, influenced by specific events and/or context [17].

Our previous longitudinal study examined a wide range of medical students’ attitudes, characteristics and experiences, including psychological variables, empathy, attitudes towards end of life care, and experience of bereavement as well as biographical details. Where possible all variables were measured using validated instruments. (See supplementary materials 1for variables examined).

The previous study was conducted in only one medical school because of this limitation we undertook a cross-sectionalcomparison of students beginning and approaching the end of their undergraduate medical education in a multicentre study, using the same survey. In addition we collected details of the timing of final examinations.

We invited all UK medical schools to participate. One aim was to examine the following questions:

1. Whether proximity of final examinations affected students’ anxiety and depression symptoms
2. Whether male and female students differed in this respect.

## Methods

Fourteen UK medical schools participated. Most participating schools provided both 5-6 year standard courses and 4 year accelerated graduate entry courses (see supplementary materials 2). It was not possible to classify schools on the basis of course style or educational delivery since accurate description of these was beyond the scope of the study. Depending on course structure, the survey was distributed to final year students either during the first or second academic term of their final year (2013/2014) and was available to them for a mean duration of 49 days. All schools sent students at least one reminder. The number and nature of reminders varied from school to school, depending on the terms of the ethical approval granted in each institution.

Students had to give their consent online before being able to access the online questionnaire. They accessed the online questionnaire using a unique randomly generated PIN, provided to each participating school by the lead research team in Cambridge. Each school then randomly allocated tokens to their students. The lead research team automatically replaced PINs with study identifiers as only this team had access to completed questionnaires.

The Cambridge team converted raw data into scales and analysed national data using IBM SPSS version 21 [32]. Ethical approval was obtained from the University of Cambridge Psychology Research Ethics Committee (application number Pre.2012.44) and from the relevant committee in each participating medical school.

We used the Hospital Anxiety and Depression Scale (HADS), divided into HADS-Dto measure depression and HADS-Ato measure anxiety [30]. HADS is a self-reportinstrument initially developed to evaluate the presence and severity of anxiety and depression in general medical populations. It is generally regarded to have good psychometric properties for the general population [33]. In studies with populations suffering from specific illnesses specificity and sensitivity of both subscales have been reported to be acceptable [34, 35]. The HADS has been widely used in the UK with members of the general population, [36] young adults, [37] undergraduates, [38] and medical students [39, 40].

For both scales we adopted the standard norms of scores of ≥8 indicated raised symptoms of depression or anxiety and scores of ≥11 indicating caseness of depression or anxiety. Because of considerations of normal distribution, we used Mann-WhitneyU to compare scores for HADS-D, t tests to compare scores for HADS-Aand chi square to compare prevalence rates of raised or caseness of both depression and anxiety. Depending on the variable distribution Pearson and Spearman correlation were used to measure associations. We measured effect size using Cohen’s D [31]. Participants with missing data were excluded from the respective analysis.

For the purpose of the analysis we divided the final year students into two groups: those who had their final exams within 2 months after participating in the survey (imminent group) and those for whom all final exams were more than 2 months away or who had taken either all or most their final exams when completing the survey (not imminent group). There were no significant differences between the two groups forming the not imminent group (see supplementary material 3). We compared students for whom all final exams were imminent with those for whom exams were more distant or who had already taken their final exams.

We also examined the extent to which age was associated with symptoms of depression and anxiety and the relationship with type of student.

## Results

In total 780 (61.9% female) final year UK medical students participated. Response rates varied between schools ranging from 7 to 68%. Because of this variability we divided the participating schools on the basis of response rate. Taking a pragmatic approach, we selected 6 schools in which response rates were greater than 30% for final year students and for which we also had details of the timing of final exams. The analyses reported here relate only to schools in this group, which comprised 446 (61.7% female) final year students with an overall response rate of 42.3%.

Characteristics of the 446 participating final year students are presented in Table 1. One hundred sixty-fourfinal year students formed the imminent group. The remaining 282 students were divided between those for whom all final exams were more than 2 months away 196 and 86 who had taken either all or most their final exams when completing the survey (see Table 2).

**Table 1:** Biographical characteristics of participants

Gender	
Female	275 (61.7%)
Males	171 (28.3%)
Type of student	
Standard course	364 (81.6%)
Graduate course	82 (18.4%)

Average age: 24.8 years (3.437) Range: 21–52 years

**Table 2:** Timing of final exams

		Frequency	Percent
Imminent	All exams within 2 months	164	36.8
Not Imminent	All exams more than 2 months away	196	43.9
	After exams (all or some)	86	19.3
	Total	446	100.0

### Depression scores (Table 3)

Both male and female students for whom final exams were imminent recorded significantly higher mean scores for depression compared to their counterparts whose final exams were more distant or who had already taken their final exams. These differences were “modest” in terms of effect size.

**Table 3:** Depression scores amongst students differing in proximity of final exams

HADS-D	All		Females		Males	
	Timing of Final Exams					
	Imminent (*n* = 164)	Not Imminent (*n* = 282)	Imminent (*n* = 99)	Not Imminent (*n* = 176)	Imminent (*n* = 65)	Not Imminent (*n* = 106)
Mean (SD) *Median*	5.80 (3.732) *5.50*	3.37 (3.103) *3.00*	5.86 (3.788) *6.00*	3.40 (3.150) *3.00*	5.71 (3.673) *5.00*	3.32 (3.038) *3.00*

Skew: 0.975 Kurtosis:0.741

All students: Mann Whitney U: U = 13,807, z = −7.136, p < 0.001, r (effect size) 0.338

Female students: Mann Whitney U: U = 5213, z = −5.556, *p* < 0.001, r (effect size) 0.335

Male students: Mann Whitney U: U = 2051.5, z = −4.459, p < 0.001, r (effect size) 0.341

### Depression prevalence (Table 4)

Among female students for whom final exams were imminent 30.3% recorded scores indicating raised levels of depression (HADS-D score ≥ 8) as compared to just over 11.4% among female students for whom final exams were more distant. Comparable figures for male students were 30.8% compared to 9.4%. For both female and male students these differences were significant.

**Table 4:** Depression prevalence amongst students differing in proximity of final exams

Levels of depression	All		Females		Males	
	Timing of Final Exams					
	Imminent (*n* = 164)	Not Imminent (*n* = 282)	Imminent (*n* = 99)	Not Imminent *n* = 176)	Imminent (*n* = 65)	Not Imminent (*n* = 106)
Normal < 8 (%)	114 (69.5%)	252 (89.4%)	69 (69.7%)	156 (88.6)	45 (69.2%)	96 (90.6%)
Raised ≥8 < 11 (%)	33 (20.1%)	18 (6.4%)	20 (20.2%)	13 (7.4%)	13 (20.0%)	5 (4.7%)
Caseness ≥11 (%)	17 (10.4%)	12 (4.3%)	10 (10.1%)	7 (4.0%)	7 (10.8%)	5 (4.7%)

Chi Square: All 28.050 *p* < 0.001; Females 15.293 *p*,0.001; Males 13.268 *p* < 0.001

### Gender differences depression

Regardless of whether final exams were imminent or not, male and female students recorded very similar mean scores and prevalence rates indicating no substantial gender difference in respect of depression symptoms (Table 3, Table 4).

### Anxiety scores (Table 5)

Male and female students for whom final exams were imminent recorded significantly higher mean scores for anxiety compared to their counterparts whose final exams were more distant or who had already taken their final exams. These differences were large in terms of effect size, particularly among male students.

**Table 5:** Anxiety scores for students differing in proximity of final exams

HADS-A	All		Females		Males	
	Timing of Final Exams					
	Imminent (n = 164)	Not Imminent (n = 282)	Imminent (n = 99)	Not Imminent (n = 176)	Imminent (n = 65)	Not Imminent (n = 106)
Mean	10.60	7.55	11.15	8.40	9.75	6.14
(SD)	(4.419)	(4.240)	(4.260)	(4.255)	(4.555)	(3.836)
*Median*	*10.00*	*7.00*	*11.0*	*8.00*	*9.00*	*5.00*

Skew: 0.280 Kurtosis: 0.587

All students: t = 7.198, *p* < 0.001; 95% ci 2.213/3.876; r (effect size) =0.7043

Female students: t = 5.139, *p* < 0.001; 95% ci 1.695/3.801; r (effect size) =0.6459

Male students: t = 5.561, p < 0.001; 95% ci 2.330/4.895; r (effect size) = 0.8573

### Anxiety prevalence (Table 6)

Among female students for whom final exams were imminent, 75.8% recorded scores indicating raised levels of anxiety (HADS-A score ≥ 8) compared with 56.8% for whom final exams were more distant (Table 6). Comparable figures for male students were 72.3 and 33.0%. For both female and male students these differences were significant.

**Table 6:** Anxiety prevalence amongst students differing in proximity of final exams

Levels of anxiety	All		Females		Males	
	Timing of Final Exams					
	Imminent (n = 164)	Not Imminent (n = 282)	Imminent (n = 99)	Not Imminent (n = 176)	Imminent (n = 65)	Not Imminent (n = 106)
Normal < 8 (%)	42 (26.6%)	146 (51.8%)	24 (24.2%)	76 (43.2%)	18 (27.7%)	70 (66.0%)
Raised ≥8 < 11 (%)	41 (25.0%)	69 (24.5%)	20 (20.2%)	46 (26.1%)	21 (32.3%)	23 (21.7%)
Caseness ≥11 (%)	81 (49.4%)	67 (23.8%)	55 (55.6%)	54 (30.7%)	26 (40.0%)	13 (12.3%)

Chi Square: All 37.380 *p* < 0.001; Female students: 17.070 *p* < 0.001; Male students: 26.866 *p* < 0.001

### Gender differences anxiety

Among students for whom exams were imminent females recorded significantly higher mean scores for anxiety than males (t = 1.999, *p* = 0.047) (Table 5). However the percentages of male and female students recording scores indicating raised levels of anxiety (HADS-A score ≥ 8) were similar, although a far higher proportion of female students recorded scores of 11 or more (Table 6).

Among students for whom exams were not imminent female students recorded significantly higher mean scores for anxiety (t = 4.484, *p* < 0.01) and a significantly higher proportion of female students recorded scores indicating raised levels of anxiety (HADS-A score ≥ 8) Chi Square 13.841 *p* < 0.001.

Age was weakly associated with higher scores for both depression and anxiety. Graduate course students as a whole were found to record significantly higher scores for depression than standard course students, but not for anxiety. However, among graduate entry students those whom exams were imminent recorded significantly higher depression and anxiety scores than their counterparts for whom exams were not imminent (see Supplementary materials 2).

## Discussion

The timing of final exams was associated with differences in depression and anxiety symptoms experienced by final year students. Those facing exams within 2 months recorded significantly higher scores for both depression and anxiety and proportionately more displayed “raised” levels of both depression and anxiety than their counterparts for whom exams were more distant or had already been taken. Female and male students appeared equally affected by the proximity of final exams.

Both the mean score and prevalence of raised levels of depression indicated by the HADS-Dscore among final year students whose final exams were not imminent or had been taken were very similar to those recorded by students in their clinical years (years 4 to 6) in our previous study who participated at the beginning of their academic year [17]. They are also similar to a large-scalegeneral population study using HADS-Din which almost a third of participants were in an age range comparable to medical students [41].

Both the mean score and prevalence of depression indicated by the HADS-Dscore among final year students whose final exams were imminent were higher than those found in our previous study but markedly below those reported in some other studies of medical students [17, 21]. However as highlighted by Hope and Henderson there are problems of comparability of instruments [7].

Unlike other studies we found no marked gender differences in respect of mean score or prevalence of raised levels of depression scores [13, 18, 19]. In respect of anxiety it is difficult to compare our findings with those of other studies of medical students since few studies have measured anxiety using HADS-A.Furthermore questions have been raised about the appropriateness of applying the same cut-offpoints as those used for depression to indicate raised levels of anxiety using HADS-A[42, 43]. Given that effect sizes of difference in mean scores recorded by students differing in respect of proximity of final exams were large, we conclude that anxiety levels in both female and male students increase. However the extent to which this is clinical relevant remains to be investigated.

The results of this study support the view found in our previous longitudinal study, that for many medical students heightened depression may be transitory [17]. To a lesser extent similar comments may be made in respect of anxiety. This is not to undermine the seriousness of depression or excessive anxiety experienced by medical students. Rather it is to suggest that by highlighting specific context or events when students’ mental health may be deteriorating may enable better and more targeted support, particularly when resources are limited. Identification of such times when and contexts may encourage medical educators to engage in more open discussions. Specifically it is important to encourage both students and educators to acknowledge openly that there may be times and experiences which affect mental health, such as exams and/or first death of a patient [44]. This may particularly relevant since recent Australian research suggests that the medical school environment may contribute more to depressive symptoms in medical students than factors in their personal lives [45]. Establishment of special groups of medical educators involved in student support such as MEDISS may foster greater openness. Further given pressures on resources provision of support may be easier at defined times.

Equally important, by emphasising that any deterioration in mental health is common at specific times and may be transitory may also encourage a more open attitude among medical students themselves towards seeking help which evidence suggests they are reluctant to do [26–29].

Currently there is considerable research focus on mental health of medical students as the pass through their training. However apart from indicating the stage in their course few studies mention the specific context in which the study is being undertaken. The study reported here suggests that this is a serious omission and needs to be addressed in future research.

### Limitations

Although our results cannot be generalised to all UK medical schools the strengths of the study reported here are firstly the number of final year medical students participants and secondly the fact that schools differed in terms of course structure and content. Although self-reportvia a questionnaire does not provide a clinical diagnosis the HADS scale does give an indication of the level symptoms of depression and anxiety. The cross-sectionalnature of the study and the fact that timing of exams was linked to schools are also weaknesses. The overall response rate to the study was low, which we sought to address by limiting our analysis to schools achieving a response rate of more than 30%. This need to restrict analysis to a small number of schools introduces the possibility of bias.

## Conclusions

This study demonstrates the negative impact of proximity of final exams on the mental health of final year medical students; female and male students alike. For medical schools it suggests that there may be times in the undergraduate medical curriculum when additional or targeted support is needed. By identifying particularly stressful times which may have negative impacts students may be encouraged to seek help without fear of embarrassment or stigmatisation. For medical education research, the study highlights the need for greater specificity of detailed context, particularly the timing of examinations, when investigating mental health.

## Supplementary information

**Additional file 1 **Survey description**.**List of measures included in the survey including their references and demographic information accessed.

**Additional file 2.**Graduate course students and Standard course students. Brief explanation of what Graduate course students and Standard course students are, Table of depression and anxiety scores for graduate course and standard course students and comparison of imminent vs. not imminent groups in graduate and standard course students. Table of the associations between depression and anxiety scores and age. Groups in graduate and standard course students.

**Additional file 3.**Grouping of students. Reason behind grouping students for analysis including anxiety and depression scores for each group and comparisons between groups.

## Data Availability

The datasets used and or analysed in this study are available from the corresponding author on reasonable request. Please contact PT (pt350@medschl.cam.ac.uk).

## Abbreviations

HADS-A: Hospital Anxiety and Depression Scale – Anxiety
HADS-D: Hospital Anxiety and Depression Scale – Depression
N: Sample size
MEDISS: Medical Educators Involved in Student Support
OSCE: Objective Structured Clinical Examination
PIN: Personal Identification Number
SD: Standard deviation
UK: United Kingdom

## Supplementary information

**Supplementary information**accompanies this paper at 10.1186/s12909-020-02029-0.
