# Supplementary material for: When investigating depression and anxiety in undergraduate medical students timing of assessment is an important factor - a multicentre cross-sectional study
Source: BMC Med Educ. 2020 Apr 23;20:125. doi: 10.1186/s12909-020-02029-0 (PMC7181528; doi:10.1186/s12909-020-02029-0)
Supplement: Supplementary file 1 — Additional file 1 Survey description.List of measures included in the survey including their references and demographic information accessed. [file 12909_2020_2029_MOESM1_ESM.docx]

Supplementary Materials 1

**Survey description**

The survey consisted of the following existing questionnaires:

| Measure | Reference |
| --- | --- |
| Hospital Anxiety and Depression Scale (HADS-D) | Zigmond AS, Snaith RP. The hospital anxiety and depression scale. Acta Psychiatrica Scandinavia. 1983;67(6):361-70. |
| Hospital Anxiety and Depression Scale (HADS- A) | Zigmond AS, Snaith RP. The hospital anxiety and depression scale. Acta Psychiatrica Scandinavia. 1983;67(6):361-70. |
| Jefferson Scale of Empathy-Student Version (JSE-S)  Davis’s Interpersonal Reactivity Index (IRI) | Hojat, M., Gonnella, J. S., Nasca, T. J., Mangione, S., Vergare, M., & Magee, M. Physician empathy: definition, components, measurement, and relationship to gender and specialty. Am J Psychiatry,2002;159(9):1563-1569.  Davis M. Measuring individual differences in empathy: evidence for a multidimensional approach. J Pers, 1983; 44(1):113-26. |
| Collett-Lester Fear of Death Scale (CLFODS-R) | Lester D, Abdel-Khalek A. The Collett-Lester Fear of Death Scale: a correction Death Stud, 2003;(27):81-85. |
| Series of questions developed by Sullivan et al. | Sullivan AM, Lakoma MD, Block SD. The status of medical education in end-of-life care: a national report. J Gen Intern Med. 2003;18:685-695. |

The following biographical characteristics were assessed:

Age, Gender, Social Class, Qualified doctor relative, English as first language, Spiritual and religious orientation, Personal experience of bereavement, together with date and relationship to deceased.
