# Supplementary material for: When investigating depression and anxiety in undergraduate medical students timing of assessment is an important factor - a multicentre cross-sectional study
Source: BMC Med Educ. 2020 Apr 23;20:125. doi: 10.1186/s12909-020-02029-0 (PMC7181528; doi:10.1186/s12909-020-02029-0)
Supplement: Supplementary file 2 — Additional file 2.Graduate course students and Standard course students. Brief explanation of what Graduate course students and Standard course students are, Table of depression and anxiety scores for graduate course and standard course students and comparison of imminent vs. not imminent groups in graduate and standard course students. Table of the associations between depression and anxiety scores and age. Groups in graduate and standard course students. [file 12909_2020_2029_MOESM2_ESM.docx]

Supplementary Materials 2

**Graduate course students and Standard course students**

Most standard course medical students in the UK enter medical school after completing secondary school and most courses are either 5 or 6 years. Typically, standard course students are aged 18-20 years depending on whether or not they have taken a year out (gap year).

Graduate course medical students enter medical school after completing a first degree. (Bachelor of Science or Arts) Typically they are aged 21 years or over. Depending on the entry requirements of the medical school or university their first degree may or may not be in a science subject. Graduate entry students may also have higher degrees (masters or doctorates). Graduate entry courses are normally 4 years.

**Table S1 Characteristics of graduate course and standard course students**

|  | Graduate Course | | | Standard Course | | |
| --- | --- | --- | --- | --- | --- | --- |
| Age | mean=28.12 SD=4.42  range (23-52years) | | | mean=24.05 SD=2.65  range (21-46years) | | |
|  |  | Timing of Final Exams | |  | Timing of Final Exams | |
| HADS-D | All | Imminent  (n=43) | Not Imminent  (n=39) | All | Imminent  (n=121) | Not Imminent  (n=243) |
| Mean  (SD)  *Median* | 5.24  (3.533)  *5.00* | 6.58  (3.567)  *6.00* | 3.77  (2.879)  *4.00* | 4.04  (3.513)  *3.00* | 5.52  (3.764)  *5.00* | 3.31  (3.139)  *3.00* |
| HADS-A |  |  |  |  |  |  |
| Mean  (SD)  Median | 9.17  (4.671)  *9.00* | 11.05  (4.293)  *12.00* | 7.10  (4.216)  *6.00* | 8.56  (4.516)  *8.00* | 10.44  (4.470)  *10.00* | 7.63  (4.248)  *7.00* |

**Comparison of HADS scores by timing of final exams (imminent vs. not imminent)**

Graduate course students:

Depression: t=3.944, p≤0.001, Mann-Whitney U=458.5, p≤0.001; Anxiety: t=4.190, p≤0.001

Standard course students:

Depression: t=5.571, p≤0.001, Mann-Whitney = 9171.5, p≤0.001; Anxiety: t=5.847, p≤0.001

**Table S2 Association between depression and anxiety scores and age:**

|  | HADS-D  Spearman correlation | | | HADS-A  Pearson correlation | | |
| --- | --- | --- | --- | --- | --- | --- |
|  | All  (n=446) | Graduate course  (n= 82) | Standard course  (n=364) | All  (n=446) | Graduate course  (n= 82) | Standard course  (n=364) |
| age | 0.136** | 0.327** | 0.019 | 0.125** | 0.210 | 0.082 |

** p ≤ .01
