# Supplementary material for: When investigating depression and anxiety in undergraduate medical students timing of assessment is an important factor - a multicentre cross-sectional study
Source: BMC Med Educ. 2020 Apr 23;20:125. doi: 10.1186/s12909-020-02029-0 (PMC7181528; doi:10.1186/s12909-020-02029-0)
Supplement: Supplementary file 3 — Additional file 3.Grouping of students. Reason behind grouping students for analysis including anxiety and depression scores for each group and comparisons between groups. [file 12909_2020_2029_MOESM3_ESM.docx]

Supplementary Materials 3

**Grouping of students**

The reason behind grouping those for whom all final exams were more than 2 months away and those who had taken either all or most their final exams when completing the survey to the not imminent groups was that there was no significant difference between both groups.

**Table S3 HADS data for imminent and not imminent group subgroups**

| Group |  | HADS-A  Anxiety score | HADS-D  Depression Score |
| --- | --- | --- | --- |
| Exams within 2 months (all or some) | Mean | 10.60 | 5.80 |
|  | N | 164 | 164 |
|  | Std. Deviation | 4.419 | 3.732 |
|  | Range | 19 | 20 |
|  | Kurtosis | -0.632 | 0.444 |
|  | Skewness | 0.034 | 0.656 |
| Exams more than 2 months away | Mean | 7.75* | 3.61** |
|  | N | 196 | 196 |
|  | Std. Deviation | 4.137 | 3.200 |
|  | Range | 20 | 16 |
|  | Kurtosis | -0.393 | 1.360 |
|  | Skewness | 0.446 | 1.242 |
| After exams (all or some) | Mean | 7.10* | 2.83** |
|  | N | 86 | 86 |
|  | Std. Deviation | 4.459 | 2.813 |
|  | Range | 18 | 11 |
|  | Kurtosis | -0.320 | 0.409 |
|  | Skewness | 0.435 | 1.053 |
| Total | Mean | 8.67 | 4.26 |
|  | N | 446 | 446 |
|  | Std. Deviation | 4.546 | 3.543 |
|  | Range | 20 | 20 |
|  | Kurtosis | -0.587 | 0.741 |
|  | Skewness | 0.280 | 0.975 |

* p = 0.74; ** p = 0.21
